# Real-world implementation and cost impact analysis of Oncotype DX testing in early-stage breast cancer

**DOI:** 10.1371/journal.pone.0353941

**Published:** 2026-07-16

**Authors:** Niina Mäenpää, Leena Tiainen, Janne Martikainen, Minna Tanner, Arja Jukkola, Maarit Bärlund

**Affiliations:** 1 Faculty of Medicine and Health Technology, Tampere University, Tampere, Finland; 2 Department of Oncology, Tays Cancer Centre, Tampere University Hospital, The Wellbeing Services County of Pirkanmaa, Tampere, Finland; 3 School of Pharmacy, University of Eastern Finland, Kuopio, Finland; Weill Cornell University, UNITED STATES OF AMERICA

## Abstract

**Background:**

The decision on appropriate adjuvant treatment for patients with hormone receptor-positive (HR+) and human epidermal growth factor receptor 2 -negative (HER2-) early breast cancer (eBC) is sometimes challenging. Genomic assays have improved individualized treatment decisions by providing information on the genomic risk of tumors. The Oncotype DX assay (ODX) is both prognostic and predictive of chemotherapy benefit. This study aimed to evaluate the implementation process of the ODX into clinical practice, the impact of ODX on adjuvant chemotherapy treatment decisions and conduct a cost impact analysis.

**Patients and methods:**

A retrospective study at Tampere University Hospital included 129 patients with HR + , HER2- eBC tested with ODX between September 2021 and September 2023. Treatment decisions as well as adjuvant chemotherapy (CT) costs, associated health provider costs, and societal costs were projected.

**Results:**

ODX testing significantly altered CT recommendations by reducing unnecessary CT for 73% of the study patients. With ODX testing, the cost impact analysis revealed partial cost savings for the health care provider due to reduced CT administration. From a societal perspective, the use of ODX resulted in substantial savings largely attributed to the maintenance of work productivity among working-aged and employed patients.

**Conclusion:**

This study highlights the importance of streamlined testing processes and the need for clinician training in interpreting test results in a real-world setting. In addition, the findings underscore the role of ODX in enhancing personalized treatment in HR + , HER2- eBC while reducing overtreatment. The cost analysis indicates substantial cost savings with ODX testing.

## Introduction

The decision on accurate adjuvant treatment for patients with hormone receptor-positive (HR+) and human epidermal growth factor receptor-negative (HER2-) early breast cancer (eBC) can be challenging. Traditionally tumor size, histologic grade, and the presence of axillary lymph node metastases combined with the patient’s menopausal status and age have guided the need for chemotherapy (CT) also in HR + , HER2- eBC. Recently, decisions on adjuvant CT have become more individual as multiple gene expression assays have been developed to provide more information on the genomic risk of the tumor and thus long-term prognosis. Recommendations for the utilization of genomic profiling have been incorporated into major international and national breast cancer guidelines [1–3].

The Oncotype DX^®^ assay (ODX) (Exact Sciences Corporation, Madison, WI, USA) provides a Recurrence Score® (RS) result between 0 and 100, which is derived from 21 specific BC-related genes [4]. ODX has been validated to be both prognostic of distant recurrence [4,5] and predictive of CT benefit [6–9]. In recent trials of CT benefit, patients with node-negative eBC had no additional benefit from CT compared with endocrine therapy (ET) with an RS result < 26, with the exception of patients ≤ 50 years old with an RS result of 16–25, who showed some benefit from CT [9]. For postmenopausal patients with 1–3 axillary lymph node metastases and a RS result of 0–25, there was no benefit of adding CT to ET. However, for premenopausal patients with 1–3 axillary lymph node metastases, a benefit was observed also with an RS result ≤ 25 [8].

With adjuvant CT, patients are predisposed to even potentially life-threatening or lifelong side effects [10]. Thus, it is crucial that adjuvant CT is directed to patients who are most likely to benefit from it. Adjuvant CT can have a widespread and long-term negative impact on quality of life, including physical, mental, and social well-being [11]. Additionally, adjuvant CT and related adverse events cause a substantial economic burden to patients, hospitals, and society. In addition, employed patients are usually on sick leave for several months during CT, leading to significant losses for both society and the individual.

Introducing ODX to the traditional workflow can be challenging. Delays to adjuvant CT might adversely affect clinical outcomes and contribute to increased patient anxiety [12,13]. Implementing ODX reflex testing and shifting the responsibility of ordering the assay to breast surgeons has significantly reduced delays of adjuvant CT decision-making and initiation [14].

In this study, we evaluated the implementation of the internal ordering process of the ODX and how testing influenced clinical treatment decisions in a university hospital. We also conducted a cost impact analysis utilizing data from patients treated for eBC and tested with ODX in our clinical practice.

### Patients and methods

We conducted a retrospective, observational real-world study including all consecutive patients with HR + , HER2- eBC who were tested with ODX at Tampere University Hospital (TaUH) between September 21, 2021, and September 20, 2023, following definitive breast surgery. During the study period 1,288 patients were treated for BC at TaUH, of whom 129 (10%) patients met the criteria for ODX testing and were included in the study. In TaUH ODX testing is integrated into routine clinical practice for selected patients.

**Fig 1:**
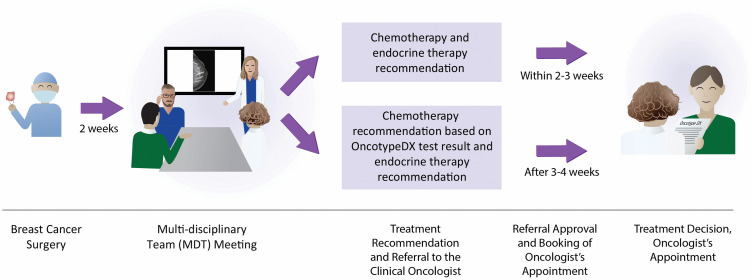
Flowchart of the introduction of Oncotype DX to early breast cancer patients’ clinical treatment process. After the pathology report is completed, an adjuvant treatment recommendation is provided for patients at the MDT meeting held twice a week at TaUH. The ordering process of ODX by the MDT aimed to minimize treatment delays and reduce the wastage of ODX by an assessment of both the patient’s tumor characteristics and clinical condition in the MDT meeting. The oncologist’s appointment was postponed for one week compared to the routine schedule to ensure that the ODX result was available and could be exploited during the appointment. The lower part illustrates the flow for patients tested with ODX and the upper part for patients without ODX testing.

The decision to perform ODX testing was made in the breast cancer multidisciplinary team (MDT) meeting. The internal criteria for ODX testing utilized in the MDT meeting are presented in Table 1. The criteria were designed to align with guideline‑supported indications while allowing clinical judgment in borderline cases. Importantly, all patients who fulfilled the criteria for ODX testing were initially considered as candidates for adjuvant CT based on conventional clinicopathological assessment prior to ODX testing in the MDT. The study patients therefore represent a clinically relevant population in which the chemotherapy decision was uncertain and the use of ODX testing was expected to inform treatment decision. The clinical workflow for the integration of ODX into decision-making is illustrated in Fig 1.

**Table 1:** Oncotype DX testing criteria. All patients were required to fulfill criteria 1-3.

Testing criteria	Comments
**1. ER or PR positive, HER2 negative breast cancer**	
**2. Clinical recurrence risk** High	High risk was defined as grade 3 tumor size ≥ 1 cm or N1; Grade 2 tumor size ≥ 2 cm or N1; Grade 1 tumor size ≥ 3 cm; and N0 or grade 1 tumor size ≥ 2 cm and N1
Low	Criteria for testing low-risk patients: Borderline tumor size compared to the high clinical risk group or multifocal tumors
**3. Menopausal status** Premenopausal patients Postmenopausal patients	pT1-2N0 pT1-2N1 only by MDT’s choice* pT1-2N0-1 pT3N0-1 (grade I-II)

*Premenopausal patients with a low RS score could be considered for ovarian function suppression + aromatase inhibitor instead of chemotherapy

In routine clinical daily practice at TaUH, three cycles of 80 mg/m^2^ docetaxel (T) and three cycles of 90 mg/m^2^ epirubicin combined with 600 mg/m^2^ cyclophosphamide (EC) is the standard adjuvant CT regimen for patients with HR+ and HER2- eBC. For elderly patients and patients with significant comorbidities, adjuvant CT of four cycles of 75 mg/m^2^ docetaxel combined with 600 mg/m^2^ cyclophosphamide (TC) can be recommended instead of T + EC. All chemotherapy regimens are delivered at three‑week intervals and supported with granulocyte colony‑stimulating factor (G‑CSF), antiemetics, and other necessary supportive medications.

Patient identification, clinicopathological characteristics and adjuvant treatment decisions were gathered from electronic patient records between January 8, 2024, and May 21, 2024. Data extraction and analysis were performed independently by two clinical oncologists (LT and NM) with expertise in breast cancer treatment. The source data is accessible to the investigators if required.

**Fig 2:**
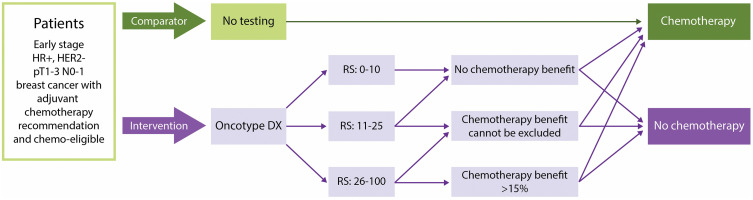
Structural overview of the cost comparison. Arrows indicate the flow of different possible treatment decisions. Abbreviations: HR = hormone receptor; HER2 = human epidermal growth factor receptor 2; RS = recurrence score.

The cost impact analysis was based on the comparison of two conceptual cohorts. In the intervention cohort, the study patients were tested with ODX, and adjuvant CT treatment decisions were made incorporating the additional information provided by the ODX test. In the comparator cohort, the same patient cohort was assumed to receive adjuvant CT without ODX testing based solely on clinical recurrence risk, reflecting treatment practices prior to the implementation of ODX testing. Fig 2 highlights the structural overview of the cost impact comparison.

In the cost impact analysis, both a public health care provider (i.e., hospital) and societal perspective were applied. Accordingly, the analysis included ODX testing costs, costs related to CT, hospitalizations due to severe acute adverse events, travel costs associated with health care visits, and productivity losses due to sick leave. As patient-level cost data were not available, unit costs were estimated using average costs for each cost component. Sensitivity analyses were performed to mitigate the inter-patient cost variability.

The unit cost of ODX was €3180 based on the manufacturer’s list price [15]. The ODX costs included the costs of all tests, including futile tests. Chemotherapy costs included the chemotherapeutic agents, preparing and administering infusions, and laboratory tests according to the hospital price tariff list. Hospital outpatient fees for chemotherapy administration and the purchase service voucher for a wig were included [16]. All treatment regimens were supported with G-CSF, antiemetics, and other necessary supportive medications to treat side effects and were captured in one price according to cost levels in 2022 [17]. Of note, patients are reimbursed for a portion of the supportive medication costs by the Finnish Social Insurance Institution, Kela. Travel costs were based on the average one-way travel allowance, which was, on average, €48 per patient in the Pirkanmaa region in 2022 [18]. Costs related to adverse events were limited to neutropenic infections requiring hospitalization. Due to the absence of local incidence data, frequency of neutropenic infections during the study period in breast cancer patients on adjuvant CT was estimated based on a Nordic adjuvant CT trial of BC patients [19]. The average length of hospitalization associated with the treatment of a neutropenic infection was estimated to be, on average, 4.6 days according to the local registry. The unit cost of treating a neutropenic infection was estimated to be €523 per inpatient hospital day [16]. Long-term adverse events and possible treatment costs of breast cancer recurrences were excluded from the present analysis due to the short time horizon of the study.

Productivity losses were estimated based on the number of working-aged women who were employed in the study population. According to the clinic’s practice, sick leave is commonly prescribed for the duration of CT treatment with an average of one month recovery time after the treatment, which was interpreted as 24 weeks for patients on T + EC chemotherapy. Through expert elicitation, the assumption was made that all employed patients were on sick leave during adjuvant chemotherapy. Based on a recent societal unit cost estimate, the monetary value of a single day of sick leave is €344 per working day for both the employer and society [20]. The number of working days was assumed to be 21 days per month (i.e., 252 working days per year).

### Statistics

Descriptive statistics were used to summarize patient and tumor characteristics. The absolute change in adjuvant CT was calculated as the difference in the proportion of patients who would have been treated with adjuvant CT without ODX testing vs. patients treated with adjuvant CT after ODX testing.

### Ethics statement

The study was approved by the local institutional review board at Tampere University Hospital (study number R23558K). Ethics Committee approval and written informed consent are not needed in single-institution register-based studies in Finland.

## Results

A total of 129 patients were tested with ODX during the study period and included in the analysis. The median age of the study population was 62 years (range 40–84 years). Sixty patients (47%) were 65 years or older, and 17 (13%) patients were over 75 years. Most patients (85%) were postmenopausal. Sixty-one (47%) of the patients were employed.

Most of the tumors were identified as grade II (74%). The median tumor size was 20 mm (range 1.8–100 mm), with 66 (51%) patients having pT1(a-c) tumors. Seventy-five (58.2%) patients had N1 nodal status, which included 26 (20.2%) patients with micro metastasis only. The patient and tumor characteristics are described in detail in Table 2.

**Table 2:** Patient characteristics.

Characteristic		N = 129 (%)
Age (years)	≤ 50	15 (12)
	> 50	114 (88)
	≥ 65	60 (47)
Menopausal status	Premenopausal	19 (15)
	Postmenopausal	110 (85)
Employment status	Employed	61 (47)
	Retired	59 (46)
	Unemployed	4 (3)
	Other	5 (4)
Histological subtype	NST	75 (58)
	Lobular	28 (22)
	Mixed	26 (20)
Histological grade	I	14 (11)
	II	96 (74)
	III	19 (15)
Tumor size	pT1a	1 (1)
	pT1b	8 (6)
	pT1c	57 (44)
	pT2	53 (41)
	pT3	10 (8)
Lymph node status	pN0	51 (39.5)
	pN (micro)	26 (20.2)
	pN1a	49 (38)
	pN2	1 (0.8)
	pNx	2 (1.5)
Clinical recurrence risk*	High	107 (83)
	Low	22 (17)
Recurrence score groups	0-10	44 (34)
	11-25	66 (51)
	> 25	19 (15)
Recommendation for chemotherapy by Oncotype Dx	Not recommended	91 (70)
	Chemotherapy benefit cannot be ruled out	23 (18)
	Chemotherapy is recommended, benefit > 15%	15 (12)

Abbreviations: pT = pathological tumor size according to TNM classification, pN = nodal involvement according to TNM classification, NST = ductal carcinoma of non-special type; *Clinical recurrence risk for hormone receptor-positive breast cancer defined as in the MINDACT trial [21] (low risk defined as well differentiated (grade I) tumors ≤ 3 cm, moderately differentiated (grade II) tumors ≤ 2 cm and poorly differentiated or undifferentiated (grade III) tumors ≤ 1 cm, node negative tumors except grade I tumors ≤ 2 cm)

The median RS was 14 (range 0–61). RS below 26 was observed in 110 (85%) patients. By the ODX result sheet, CT was not recommended for 91 (70%) patients, CT was recommended for 15 (12%) patients, and CT benefit could not be ruled out for 23 (18%) patients (Table 2).

The median duration from surgery to MDT meeting was 15 days (range 8–41 days). The MDT meeting recommended CT for all patients; a T + EC regimen for 107 patients (83%), and a TC regimen for the rest, if supported by ODX. Only 3% of the ODX were ordered futile by the MDT meeting as the test result would not have affected the treatment decision due to patient refusal or ineligibility for CT. The median delay from an MDT meeting to an ODX order was 6 days (range 1–49 days).

Overall, 34 (26%) patients underwent adjuvant CT while 91 (71%) patients omitted CT. The remaining 3% refused or were ineligible for adjuvant CT. Of the 34 patients treated with adjuvant CT, 31 patients had clinically high-risk tumors and three clinically low-risk tumors. According to tumor grade, two patients with grade I tumors received adjuvant CT. Both were postmenopausal and had RS 11–25. Among patients with grade II tumors, 23 received adjuvant CT, 15 patients with RS 11–25 and eight patients with RS > 25. All except one of these patients were postmenopausal. The premenopausal patient had a RS > 25. Nine patients with grade III tumors received adjuvant CT; one had RS 11–25 and eight had RS > 25. All adjuvant CT treated patients with grade III tumors were postmenopausal. Of the patients who had CT, nine patients were treated with the TC regimen and 25 patients with the T + EC regimen. Only eleven out of 61 patients who were employed had adjuvant CT, and everyone was treated with the T + EC regimen. The TC regimen was recommended generally for older patients, the median age being 77 years (range 60–84 years), and T + EC was recommended for younger patients, with the median age being 59.5 years (range 40–84 years).

Generally, RS will directly guide treatment recommendations and an RS of < 26 for a postmenopausal patient would support endocrine treatment without CT, whereas an RS of > 25 would justify CT. However, 17 (13%) postmenopausal breast cancer patients out of 129 study participants were treated with adjuvant CT although the RS was less than 26. The above treatment decisions were made throughout the study period by several oncologists (n = 9), including specialists and residents after consulting a specialist. The RS range for the previously noted patients was 15–25.

Of the 129 patients 107 (83%) had clinically high-risk tumors and 22 (17%) had low-risk tumors. The patients with low-risk tumors were generally younger (median age 50 years, range 40–78 years). ODX was ordered according to the prespecified testing criteria (Table 1) for 124 (96%) patients. Five patients were tested outside the testing criteria by MDT´s clinical judgment. These cases included postmenopausal patients with gr I-II tumors and isolated tumor cells or micro metastases in the lymph nodes. No specific trend was seen in the ordering practice of ODX during the study period.

The median duration from ordering an ODX to an accessible test result was 14 days (range 9–35 days). The test result was available at the time of the oncologist’s appointment for 103 (80%) patients. Thus, for 26 (20%) patients the adjuvant treatment decision was postponed until the test result was available. For patients who received adjuvant CT, the median time from breast cancer surgery to the initiation of adjuvant CT was 51.5 days (range 40–66 days). The delay from surgery to the beginning of adjuvant CT was similar throughout the study period and concentrated around holidays. For nine patients, the delay from surgery was over eight weeks, but the treatment began for all within 10 weeks after surgery.

Of the 34 patients who received adjuvant CT, treatment was interrupted for six (18%) patients due to adverse events. All these patients were older, with a median age of 60 (range 60–79), and interruptions occurred during both TC and T + EC regimens. The treatment was interrupted for various reasons, e.g., an accidental humerus fracture, exacerbation of neuropathic symptoms, mild cardiac adverse events, an overall decline in performance, and non-neutropenic or mild neutropenic infections.

### Cost impact analysis

In the cost impact analysis, two conceptual cohorts were compared as presented in Fig 2. In the overall study population, four patients were not eligible for adjuvant CT due to the comorbidities or refusal of adjuvant chemotherapy treatment. Thus, the comparator arm analysis was made according to 125 patients.

**Fig 3:**
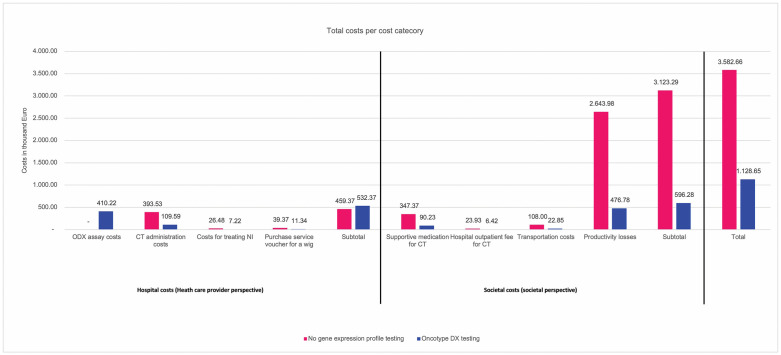
Economic impact of Oncotype DX testing. If no gene profile testing was used, 125 patients would have been treated with chemotherapy. With ODX testing, adjuvant chemotherapy was reduced by 73% and only 34 patients received chemotherapy. The most common severe adverse event related to chemotherapy is neutropenic infection, which occurs in 9% of chemotherapy-treated patients [19]. In total, using ODX testing resulted in cost savings of €2,454,000. Abbreviations: ODX = Oncotype DX; CT = chemotherapy; NI = neutropenic infection.

The results of the cost impact analysis are presented in Fig 3 and S1 Table. The implementation of ODX increased the total costs when measuring costs from the health care provider’s perspective. The total costs for the healthcare provider were €532,370 in the intervention cohort and €459,374 in the comparator cohort. Thus, the overall increase in healthcare provider costs was inflicted by the ODX costs, which exceeded the expenses of costs saved on CT administration. However, when the perspective was broadened to include all relevant cost drivers from the societal perspective, including supportive medication, hospital outpatient fees, travel costs, and productivity losses due to sick leave, the total costs in the intervention cohort were substantially lower than in the comparator cohort, €1.1 million and €3.6 million, respectively, leading to potential savings of €2.5 million. Fig 3 further highlights the economic impact of the implementation of ODX on clinical practice as a cost saving procedure.

In the sensitivity analysis, varying the net reduction of chemotherapy with ODX testing and different cost variables by ± 25% were analyzed (Table 3). Consistent cost savings were demonstrated with ODX testing despite the varied net reduction of CT and different cost variables. Chemotherapy-related costs were captured into one price and calculated with the cost of the T + EC regimen due to its predominance in adjuvant CT.

**Table 3:** Sensitivity analyses with varying net reduction of chemotherapy and cost variables by ± 25%.

Assumption category	Base case	Sensitivity tests	Sensitivity test assumptions	Estimated cost impact EUR
Net reduction in chemotherapy	73%	+25%	91%	−2 911 485
		−25%	55%	−1 977 144
Cost per Oncotype DX test	€3180	+25%	€3 975	−2 352 682
		−25%	€2 385	−2 557 792
Chemotherapy treatment-related costs*	€6743	+25%	€8 429	−2 607 774
		−25%	€5 057	−2 301 517
Productivity losses	€344	+25%	€430	−2 995 816
		−25%	€258	−1 912 216

*Chemotherapy treatment-related costs include the costs of T + EC chemotherapy, supportive medication for T + EC chemotherapy, a purchase service voucher for a wig, and hospital outpatient fee of chemotherapy administration

## Discussion

To the best of our knowledge, this is the first study ever conducted in Finland evaluating a real-world implementation process of ODX and clinical impact as well as the cost impact of ODX testing in patients diagnosed with HR + , HER2- eBC. The main finding of this study is that ODX changed the adjuvant CT recommendation for 73% of the study patients, for whom adjuvant CT treatment could be safely omitted based on ODX testing.

The results of the cost impact analysis indicate that the use of ODX increased the costs for the health care provider by almost €73,000 (16%) during the study period. By avoiding unnecessary adjuvant CT, the ODX costs were partially offset by the saved CT treatment costs. However, when the perspective was broadened to cover all relevant societal costs, ODX testing brought substantial savings worth €2.5 million during the study period. The largest proportion of the savings was attributed to the reduced sick leave and maintenance of work productivity among the working aged, employed patients who omitted CT. The results of this study are in line with the previous studies reporting the reduced administration of chemotherapy and cost-effectiveness of ODX testing [22–24]. When considering the costs from the national perspective, a broad implementation of ODX testing could lead to significant societal savings in Finland. For example, in 2022, a total of 4867 breast cancers were diagnosed in Finland [25]. Since approximately 10% of all breast cancer patients in Finland are treated at TaUH, it can be projected that the expected total savings would be approximately tenfold, i.e., over €25 million in a similar timeline.

As noted, ODX testing increases treatment costs in patients with ER + , HER2- eBC. To minimize the ODX expenses, there are advantages to guiding the decision for ODX testing to the MDT instead of reflex testing. In the MDT, patients’ clinical conditions are evaluated before ODX testing to avoid testing patients that might otherwise be ineligible for chemotherapy. During the study period, only 3% of the ODX tests were ordered unnecessarily, as patient preferences about the adjuvant CT were not known in advance or the performance status of the patient was considered better by the MDT than by evaluation of the oncologist.

MDT-based ODX testing enhances individuality and equality in eBC treatment as patients are not excluded from testing according to, e.g., age criteria. For example, in Finland 55% of eBC patients are 65 years or older [25]. The prespecified testing criteria are important in guiding the gene profile assay testing to the right patients, i.e., to the patient population in which ODX is validated. However, some patients are marginally excluded from testing. In our real-world patient population, some clinically high-risk tumors were tested in patients with borderline performance status for adjuvant CT in the hope of a low RS result to ease the clinician’s decision to omit chemotherapy. In addition, some borderline clinically low-risk patients were tested according to clinical judgment.

Surprisingly, 17 (13%) of postmenopausal eBC patients out of the 129 study participants were treated with adjuvant chemotherapy although the RS was less than 26. Previously, the RxPONDER study results were incorporated into the result sheet on a separate page, and with an unthorough review of the report or review by clinicians unfamiliar with the result, the report could mistakenly recommend unnecessary adjuvant chemotherapy. It is also possible that administration of adjuvant CT to patients with RS < 26 reflected limited confidence in, or cautious interpretation of, the ODX during the earlier phase of its implementation, and underscores that ODX results were used as one component of an integrated clinical assessment rather than as a standalone determinant of treatment. This highlights the need for training clinical oncologists in interpreting the ODX results to fully utilize the test results in adjuvant CT recommendations.

Implementing ODX in clinical practice has been associated with a more than seven-fold increase in the likelihood of delays to adjuvant CT initiation [26]. The introduction of criteria for the surgeon-initiated reflex ODX testing has led to clinically significant reductions in delays of CT initiation [14]. In the present study, the median time from breast surgery to adjuvant chemotherapy was 51.5 days. According to national guidelines, adjuvant chemotherapy should be started within 42 days of the surgery [27]. In our study, only 9% of patients received treatment without delays. This emphasizes the need to monitor the ODX introduction process in real time to minimize delays. For example, this study revealed a surprising delay in ordering ODX, which should be addressed. Of note, as a future prospect there are ongoing studies of Oncotype DX testing in the neoadjuvant setting that set further requirements for a fluent and fast testing process.

There were some limitations in this study. The patient selection was exposed to selection bias as the patients who were tested with ODX were prescreened by the MDT. Also, substantially more grade I-II tumors than grade III tumors and also borderline clinically high-risk tumors were tested which have a greater likelihood of lower RS. The time horizon of the study was short with an inadequate follow-up time to evaluate the occurrence or treatment costs of any long-term adverse effects of adjuvant CT. Additionally, due to the short time horizon of the study period, possible BC recurrences or secondary malignancies and the expenses related to their management were not included in the cost impact analysis. Adjuvant CT can cause a large spectrum of serious adverse effects with added hospital expenses, as indicated also by the study results. In this study, the only adverse effect considered was severe neutropenic infections, which are quite common among CT treated patients, and it was possible to estimate the cost of treating a neutropenic infection in a relatively reliable way based on the current treatment practices. However, other health care utilization during treatment was not captured in the analysis, which may influence the overall cost estimates. Also, as the data on the portion of the employed patients who were on sick leave during adjuvant chemotherapy were not available, the productivity loss costs due to sick leave were calculated based on the assumption that all employed patients were on sick leave. Although the former is the reality for the majority of the patients in the clinic, employment status, the duration of sick leave, ability to work during treatment, and return-to-work timelines may vary considerably between patients, and the assumption could affect the productivity loss costs for society. Finally, as no patient-level cost data were available, the cost impact analysis was performed using average costs for each cost component. Although the sensitivity analyses of the economic impact were performed to address the limitation of patient-level cost variability and supported consistent cost savings with ODX testing despite varying cost levels, the estimated costs and savings should be interpreted within the framework of the assumptions made in the cost impact analysis.

This study was the first insight regarding the cost impact of ODX testing in public health care in Finland. Future studies are warranted on the subject with more detailed health economical evaluation of the long-term costs for health care and on the benefits for patients.

## Conclusion

In routine MDT-based clinical practice, utilization of ODX enhances personalized treatment in patients with ER + , HER2- eBC, and it can substantially ease the clinical and financial burden. The wide introduction of ODX in clinical practice can lead to substantial savings for society. The process of implementing ODX in clinical practice needs monitoring to avoid delays in treatment and futile ODX testing. Clinicians need training in interpreting the ODX result.

## Supporting information

S1 Table Costs of the comparator cohort and the intervention cohort. (DOCX)
