# Supplementary material for: Real-world implementation and cost impact analysis of Oncotype DX testing in early-stage breast cancer
Source: PLoS One. 2026 Jul 16;21(7):e0353941. doi: 10.1371/journal.pone.0353941 (PMC13374984; doi:10.1371/journal.pone.0353941)
Supplement: S1 Table — (DOCX) [file pone.0353941.s001.docx]

**S1 Table**. **Costs of the comparator cohort and the intervention cohort.**

|  | Unit Cost, EUR | Source | Cost of the comparator cohort EUR (N) | Cost of the intervention cohort EUR (N) |
| --- | --- | --- | --- | --- |
| Assay costs |  |  |  |  |
| Oncotype DX | 3180 | (15) | 0 (0) | 410 220 (129) |
| Hospital costs |  |  |  |  |
| Chemotherapy |  |  |  |  |
| T+EC  TC | 3285  2385 | (16)  (16) | 348 210 (106)  45 315 (19) | 88 125 (25)  21 465 (9) |
| Treating severe neutropenic infection | 2407.20 | (16,19) | 26 479.20 (11) | 7 222 (3) |
| Purchase service voucher for a wig | 314.96 | (16) | 39 370 (125) | 11 338.50 (34) |
| Subtotal (health care provider perspective) |  |  | **459 374** | **532 370** |
| Societal costs |  |  |  |  |
| Supportive medication with chemotherapy |  |  |  |  |
| T+EC  TC | 2 947.90  1 836.70 | (17)  (17) | 312 477.40 (106)  34 897.30 (19) | 73 697.50 (25)  16 530.30 (9) |
| Hospital outpatient fee of chemotherapy |  |  |  |  |
| T+EC, N  TC, N | 195  171.80 | (16)  (16) | 20 670 (106)  3 264.20 (19) | 4875 (25)  1546.20 (9) |
| Transportation costs | 96 | (18) | 108 000 (125) | 22 848 (34) |
| Sick leave costs | 344 | (20) | 2 643 984 (61) | 476 784 (11) |
| Subtotal (societal perspective) |  |  | 3 123 292.90 | 596 281 |
| Total |  |  | **3 582 666.90** | **1 128 651** |
| Incremental cost difference |  |  |  | **-2 454 015.90** |

Abbreviations: N=number of patients
