# Supplementary material for: Real-world implementation and cost impact analysis of Oncotype DX testing in early-stage breast cancer
Source: PLoS One. 2026 Jul 16;21(7):e0353941. doi: 10.1371/journal.pone.0353941 (PMC13374984; doi:10.1371/journal.pone.0353941)
Supplement: Attachment — Submitted filename: Response to Reviewers.docx [file pone.0353941.s004.docx]

Dear Editor Bhattacharya,

We thank You for Your interest in our manuscript and the possibility of submitting a revised version of our manuscript “Real-world implementation and cost impact analysis of Oncotype DX testing in early-stage breast cancer”.

We would also like to thank the Reviewers for their careful evaluation and highly constructive and useful comments. We believe that we have properly addressed the comments of the Reviewers.

Please find enclosed the Response to Reviewers and a revised version of our manuscript. All changes made to the manuscript are highlighted in the revised version. Hopefully, the manuscript now fulfills Your requirements and qualifies for publication in PLOS One.

Sincerely,

Niina Mäenpää, MD

niina.maenpaa@tuni.fi

**Response to Reviewers**

**Reviewer #1:**

The study, like the TAILORx study, suffers from a major issue. This is that a control group is missing that was treated without receiving an Oncotype testing but assuming that an ODx score was present. This control group would show that
a) Oncotype testing is essential rather than being a tool that can be used add on and overtakes a medical decision, that may also be made if a treained team of pathologists and senologists work together and bring in their experience in concert with cheaper diagnostic tools (IHC and classical stainings combined with clinical observatins).
b) oncotype Dx is more than a diagnostic placebo
c) Oncotype Dx is indeed cost efficient, because if A9 would manifest and it should become clear that Oncotype testing can be replaced by classical methods even the 3180 € can be saved, which would also have a remarkable effect on health economics.General comment on the lack of control group

**Authors reply:**

We appreciate the reviewer’s concern regarding the absence of a non‑Oncotype DX (ODX) control group. However, we respectfully emphasize that the primary aim of our study was not to further validate the clinical utility of the ODX assay per se, but rather to describe the assay’s real‑world implementation process into our routine multidisciplinary team (MDT) practice in our clinic, the clinical impact on treatment decisions, and economic consequences of ODX testing.

All patients included in our study were initially assessed in the MDT and were considered candidates for adjuvant chemotherapy (CT) based on conventional clinicopathological factors. The reviewer’s suggestion about including a control group in our study, which would not be tested with ODX, would require a prospective randomized study design. As ODX has already been incorporated into our healthcare system and clinical practice and acknowledged in major international breast cancer guidelines, it would be neither ethically nor practically feasible to recruit patients in such a study. As previously noted, further validation of the ODX was not in the scope of this real‑world observational study.

Importantly, contrary to the assumption that ODX might simply replace clinical judgment in adjuvant treatment decisions, our findings demonstrate that treatment decisions were made within the MDT framework with established pathological and clinical variables reinforced by genomic information of the ODX test result. Moreover, in our study also patients with low recurrence scores (RS) received adjuvant chemotherapy, which suggests that ODX did not function as an automatic or decisive determinant of care but rather reflected limited confidence in, or cautious interpretation of, the ODX assay during the early phase of its implementation. This issue has been further discussed in the Discussion section (revised manuscript page 15).

We respectfully disagree with the characterization of ODX as a “diagnostic placebo.” Our economic analysis was conducted using real treatment decisions and observed chemotherapy avoidance, not hypothetical scenarios. The resulting cost estimates therefore reflect actual resource utilization in routine care. We have revised the Methods section to better detail the assumptions used in cost calculations, as requested also by Reviewer #2.

Regarding the reviewer’s mention of “A9”, the concept was unclear (maybe a typographical error?) and we didn’t address this specifically. We have ensured that the economic rationale is now presented more transparently and grounded in observed data rather than speculative alternatives.

**Reviewer comment:**

The study has no added value which is typical for the majority of clinical Oncotype studies for which at least one author have received speaker honorary from Exact Sciences or predecessor organisations.

**Authors reply:**

We appreciate the reviewer’s concern about the clinical value of our study. We strongly believe that the added value of the present study lies in:

- The real‑world, population‑based data from routine MDT practice
- Implementation analysis in a publicly funded healthcare system;
- Economic impact assessment reflecting actual clinical pathways rather than trial‑based assumptions.

We believe these aspects are highly relevant to clinicians and policymakers and justify publication independently of previous randomized trials such as TAILORx.

We appreciate the reviewer’s comment regarding the potential conflicts of interest. We would like to clarify that one of the authors received a speaker’s honorarium from Exact Sciences for a single lecture held in January 2023, at which time ODX testing was already established in our clinical practice. The remaining authors have no associations with Exact Sciences, and all other potential financial interests have been disclosed in full.

**Reviewer #2:**

**Reviewer comment:**

The manuscript is well written with clear ideas and addresses an important topic on the clinical and economic impact of Oncotype DX testing. However, methodological details and patient selection criteria should be clarified.

**Authors reply:**

We thank the reviewer for this helpful suggestion. We have expanded the Patients and Methods section (revised manuscript page 4) to clarify the patient selection criteria, the clinical context of the study, and the different scenarios of the cost impact analysis.

**Reviewer comment:**

The economic analysis is useful, but the assumptions used in cost calculations should be explained more clearly.

**Authors reply:**

We thank the reviewer for the excellent comment. As there were no patient-level cost data available, the cost units were estimated by average costs for each cost category. Given the inherent variability in patient-level costs, we conducted a sensitivity analysis to account for this variation.

We have added clarification based on the comment (Patients and Methods, revised manuscript page 6) to improve the reproducibility and interpretability of the economic findings and discussed the topic in the Discussion section (revised manuscript page 15). Of note, the cost calculations are presented in more detail in the supplementary material S1 table.

**Reviewer comment:**

I recommend adding a schematic figure of the Oncotype DX assay (21-gene panel) or workflow would improve understanding of the test.

**Authors reply:**

We appreciate the reviewer’s suggestion. Given that Oncotype DX is a widely used and well‑established commercial assay, we chose not to include detailed gene‑level information of the assay to maintain the manuscript’s focus on the main topics such as the clinical implementation of the test rather than the molecular test itself. However, we have ensured that appropriate references are cited describing the assay for readers who wish to explore the assay in greater detail.

**Reviewer comment:**

I also recommend analysing the relationship between tumor grade and treatment decisions based on the Oncotype DX recurrence score.

**Authors reply:**

We thank the reviewer for this insightful suggestion. We performed this additional analysis and included the results in the revised manuscript (Results, revised manuscript page 10).
