# Supplementary material for: Real-world implementation and cost impact analysis of Oncotype DX testing in early-stage breast cancer
Source: PLoS One. 2026 Jul 16;21(7):e0353941. doi: 10.1371/journal.pone.0353941 (PMC13374984; doi:10.1371/journal.pone.0353941)
Supplement: Attachment — Submitted filename: Response_to_Reviewers_auresp_2.docx [file pone.0353941.s005.docx]

Dear Editor Bhattacharya,

We thank You for Your thoughtful evaluation of our revised manuscript and for the opportunity to submit a further revised version of our manuscript “Real-world implementation and cost impact analysis of Oncotype DX testing in early-stage breast cancer.”

We are grateful for the constructive comments provided during the review process, which have helped us to further improve the clarity of the manuscript. In this second revision, we have addressed the remaining points raised in the Discussion, with particular attention to clarifying the interpretation of the economic analysis and explicitly acknowledging the assumptions and uncertainties underlying the cost impact estimates.

All changes made to the manuscript are highlighted in the revised version. We believe that these revisions have further strengthened the manuscript.

We hope that the manuscript now meets Your requirements for publication in PLOS One.

Sincerely,
Niina Mäenpää, MD
niina.maenpaa@tuni.fi
