# Supplementary material for: Real-world implementation and cost impact analysis of Oncotype DX testing in early-stage breast cancer
Source: PLoS One. 2026 Jul 16;21(7):e0353941. doi: 10.1371/journal.pone.0353941 (PMC13374984; doi:10.1371/journal.pone.0353941)
Supplement: Peer review history

# Peer review: Decision Letter 0

**DOI:** 10.1371/journal.pone.0353941.r001

**Authors:** UDAYAN BHATTACHARYA

25 Mar 2026

PONE-D-25-55193

Real-world implementation and cost impact analysis of Oncotype DX testing in early-stage breast cancer

PLOS One

Dear Dr. Mäenpää,

Thank you for submitting your manuscript to PLOS ONE. After careful consideration, we feel that it has merit but does not fully meet PLOS ONE’s publication criteria as it currently stands. Therefore, we invite you to submit a revised version of the manuscript that addresses the points raised during the review process.

Please submit your revised manuscript by May 09 2026 11:59PM. If you will need more time than this to complete your revisions, please reply to this message or contact the journal office at plosone@plos.org. When you're ready to submit your revision, log on to https://www.editorialmanager.com/pone/ and select the 'Submissions Needing Revision' folder to locate your manuscript file.

Please include the following items when submitting your revised manuscript:

- A letter that responds to each point raised by the academic editor and reviewer(s). You should upload this letter as a separate file labeled 'Response to Reviewers'.
- A marked-up copy of your manuscript that highlights changes made to the original version. You should upload this as a separate file labeled 'Revised Manuscript with Track Changes'.
- An unmarked version of your revised paper without tracked changes. You should upload this as a separate file labeled 'Manuscript'.

If you would like to make changes to your financial disclosure, please include your updated statement in your cover letter. Guidelines for resubmitting your figure files are available below the reviewer comments at the end of this letter.

If applicable, we recommend that you deposit your laboratory protocols in protocols.io to enhance the reproducibility of your results. Protocols.io assigns your protocol its own identifier (DOI) so that it can be cited independently in the future. For instructions see: https://journals.plos.org/plosone/s/submission-guidelines#loc-laboratory-protocols. Additionally, PLOS ONE offers an option for publishing peer-reviewed Lab Protocol articles, which describe protocols hosted on protocols.io. Read more information on sharing protocols at https://plos.org/protocols?utm_medium=editorial-email&utm_source=authorletters&utm_campaign=protocols.

As the corresponding author, your ORCID iD is verified in the submission system and will appear in the published article. PLOS supports the use of ORCID, and we encourage all coauthors to register for an ORCID iD and use it as well. Please encourage your coauthors to verify their ORCID iD within the submission system before final acceptance, as unverified ORCID iDs will not appear in the published article. *Only*  the individual author can complete the verification step; PLOS staff *cannot*  verify ORCID iDs on behalf of authors.

We look forward to receiving your revised manuscript.

Kind regards,

UDAYAN BHATTACHARYA

Academic Editor

PLOS One

Journal Requirements:

When submitting your revision, we need you to address these additional requirements.

1. Please ensure that your manuscript meets PLOS ONE's style requirements, including those for file naming. The PLOS ONE style templates can be found at

https://journals.plos.org/plosone/s/file?id=wjVg/PLOSOne_formatting_sample_main_body.pdf and

https://journals.plos.org/plosone/s/file?id=ba62/PLOSOne_formatting_sample_title_authors_affiliations.pdf

2. We note that the grant information you provided in the ‘Funding Information’ and ‘Financial Disclosure’ sections do not match.

When you resubmit, please ensure that you provide the correct grant numbers for the awards you received for your study in the ‘Funding Information’ section.

3. Thank you for stating the following in the Competing Interests section: [I have read the journal's policy and the authors of this manuscript have the following competing interests: LT has received speakers' bureau honoraria from Exact Sciences. The company was not involved carrying out this study. JM is a founding partner of ESiOR Oy, a health economic analytics company. The company was not involved in carrying out this research.].

Please confirm that this does not alter your adherence to all PLOS ONE policies on sharing data and materials, by including the following statement: ""This does not alter our adherence to  PLOS ONE policies on sharing data and materials.” (as detailed online in our guide for authors http://journals.plos.org/plosone/s/competing-interests).  If there are restrictions on sharing of data and/or materials, please state these. Please note that we cannot proceed with consideration of your article until this information has been declared.

Please include your updated Competing Interests statement in your cover letter; we will change the online submission form on your behalf.

4. If the reviewer comments include a recommendation to cite specific previously published works, please review and evaluate these publications to determine whether they are relevant and should be cited. There is no requirement to cite these works unless the editor has indicated otherwise.

Additional Editor Comments:

Please answer to the reviewer's questions point by point and resubmit

[Note: HTML markup is below. Please do not edit.]

Reviewers' comments:

Reviewer's Responses to Questions

**Comments to the Author**

1. Is the manuscript technically sound, and do the data support the conclusions?

The manuscript must describe a technically sound piece of scientific research with data that supports the conclusions. Experiments must have been conducted rigorously, with appropriate controls, replication, and sample sizes. The conclusions must be drawn appropriately based on the data presented.

Reviewer #1: No

Reviewer #2: Yes

**********

2. Has the statistical analysis been performed appropriately and rigorously?

Reviewer #1: No

Reviewer #2: Yes

**********

3. Have the authors made all data underlying the findings in their manuscript fully available?

The PLOS Data policy requires authors to make all data underlying the findings described in their manuscript fully available without restriction, with rare exception (please refer to the Data Availability Statement in the manuscript PDF file). The data should be provided as part of the manuscript or its supporting information, or deposited to a public repository. For example, in addition to summary statistics, the data points behind means, medians and variance measures should be available. If there are restrictions on publicly sharing data—e.g. participant privacy or use of data from a third party—those must be specified.

Reviewer #1: Yes

Reviewer #2: Yes

**********

4. Is the manuscript presented in an intelligible fashion and written in standard English?

PLOS ONE does not copyedit accepted manuscripts, so the language in submitted articles must be clear, correct, and unambiguous. Any typographical or grammatical errors should be corrected at revision, so please note any specific errors here.

Reviewer #1: Yes

Reviewer #2: Yes

**********

5. Review Comments to the Author

Please use the space provided to explain your answers to the questions above. You may also include additional comments for the author, including concerns about dual publication, research ethics, or publication ethics. (Please upload your review as an attachment if it exceeds 20,000 characters)

Reviewer #1: The study, like the TAILORx study, suffers from a major issue. This is that a control group is missing that was treated without receiving an Oncotype testing but assuming that an ODx score was present. This control group would show that

a) Oncotype testing is essential rather than being a tool that can be used add on and overtakes a medical decision, that may also be made if a treained team of pathologists and senologists work together and bring in their experience in concert with cheaper diagnostic tools (IHC and classical stainings combined with clinical observatins).

b) oncotype Dx is more than a diagnostic placebo

c) Oncotype Dx is indeed cost efficient, because if A9 would manifest and it should become clear that Oncotype testing can be replaced by classical methods even the 3180 € can be saved, which would also have a remarkable effect on health economics.

Thus, the study has no added value which is typical for the majority of clinical Oncotype studies for which at least one author have received speaker honorary from Exact Sciences or predecessor organisations.

However, I would would reconsider the study if a proper control group will be included.

Reviewer #2: The manuscript is well written with clear ideas and addresses an important topic on the clinical and economic impact of Oncotype DX testing. However, methodological details and patient selection criteria should be clarified.

The economic analysis is useful, but the assumptions used in cost calculations should be explained more clearly.

I recommend adding a schematic figure of the Oncotype DX assay (21-gene panel) or workflow would improve understanding of the test.

I also recommend analysing the relationship between tumour grade and treatment decisions based on the Oncotype DX recurrence score.

**********

6. PLOS authors have the option to publish the peer review history of their article (what does this mean?). If published, this will include your full peer review and any attached files.

If you choose “no”, your identity will remain anonymous but your review may still be made public.

**Do you want your identity to be public for this peer review?**  For information about this choice, including consent withdrawal, please see our Privacy Policy.

Reviewer #1: No

Reviewer #2: No

**********

[NOTE: If reviewer comments were submitted as an attachment file, they will be attached to this email and accessible via the submission site. Please log into your account, locate the manuscript record, and check for the action link "View Attachments". If this link does not appear, there are no attachment files.]

To ensure your figures meet our technical requirements, please review our figure guidelines: https://journals.plos.org/plosone/s/figures

You may also use PLOS’s free figure tool, NAAS, to help you prepare publication quality figures: https://journals.plos.org/plosone/s/figures#loc-tools-for-figure-preparation.

NAAS will assess whether your figures meet our technical requirements by comparing each figure against our figure specifications.

Attachment Submitted filename: PONE-D-25-55193.pdf

# Author comment: Author response to Decision Letter 1

**DOI:** 10.1371/journal.pone.0353941.r002

29 May 2026

To follow up the revised version of our manuscript, in the journal requirements it was asked to check the grant numbers of the funders of our study. I have rechecked the funding numbers and they are stated correctly in the "Funding information" section.

The Reviewers comments are answered point-by-point in the "Response to Reviewers" file.

Attachment Submitted filename: Response to Reviewers.docx

# Editor report: Decision Letter 1

**DOI:** 10.1371/journal.pone.0353941.r003

**Authors:** UDAYAN BHATTACHARYA, UDAYAN BHATTACHARYA

1 Jun 2026

PONE-D-25-55193R1

Real-world implementation and cost impact analysis of Oncotype DX testing in early-stage breast cancer

PLOS One

Dear Dr. Mäenpää,

Thank you for submitting your manuscript to PLOS ONE. After careful consideration, we feel that it has merit but does not fully meet PLOS ONE’s publication criteria as it currently stands. Therefore, we invite you to submit a revised version of the manuscript that addresses the points raised during the review process.

Please submit your revised manuscript by Jul 16 2026 11:59PM. If you will need more time than this to complete your revisions, please reply to this message or contact the journal office at plosone@plos.org. When you're ready to submit your revision, log on to https://www.editorialmanager.com/pone/ and select the 'Submissions Needing Revision' folder to locate your manuscript file.

Please include the following items when submitting your revised manuscript:

- A letter that responds to each point raised by the academic editor and reviewer(s). You should upload this letter as a separate file labeled 'Response to Reviewers'.
- A marked-up copy of your manuscript that highlights changes made to the original version. You should upload this as a separate file labeled 'Revised Manuscript with Track Changes'.
- An unmarked version of your revised paper without tracked changes. You should upload this as a separate file labeled 'Manuscript'.

If you would like to make changes to your financial disclosure, please include your updated statement in your cover letter. Guidelines for resubmitting your figure files are available below the reviewer comments at the end of this letter.

If applicable, we recommend that you deposit your laboratory protocols in protocols.io to enhance the reproducibility of your results. Protocols.io assigns your protocol its own identifier (DOI) so that it can be cited independently in the future. For instructions see: https://journals.plos.org/plosone/s/submission-guidelines#loc-laboratory-protocols. Additionally, PLOS ONE offers an option for publishing peer-reviewed Lab Protocol articles, which describe protocols hosted on protocols.io. Read more information on sharing protocols at https://plos.org/protocols?utm_medium=editorial-email&utm_source=authorletters&utm_campaign=protocols.

As the corresponding author, your ORCID iD is verified in the submission system and will appear in the published article. PLOS supports the use of ORCID, and we encourage all coauthors to register for an ORCID iD and use it as well. Please encourage your coauthors to verify their ORCID iD within the submission system before final acceptance, as unverified ORCID iDs will not appear in the published article. *Only*  the individual author can complete the verification step; PLOS staff *cannot*  verify ORCID iDs on behalf of authors.

We look forward to receiving your revised manuscript.

Kind regards,

UDAYAN BHATTACHARYA

Academic Editor

PLOS One

**Journal Requirements:**

1. If the reviewer comments include a recommendation to cite specific previously published works, please review and evaluate these publications to determine whether they are relevant and should be cited. There is no requirement to cite these works unless the editor has indicated otherwise.

2. Please review your reference list to ensure that it is complete and correct. If you have cited papers that have been retracted, please include the rationale for doing so in the manuscript text, or remove these references and replace them with relevant current references. Any changes to the reference list should be mentioned in the rebuttal letter that accompanies your revised manuscript. If you need to cite a retracted article, indicate the article’s retracted status in the References list and also include a citation and full reference for the retraction notice.

**Additional Editor Comments:**

Dear Authors,

Thank you for submitting your manuscript, "Real-world implementation and cost impact analysis of Oncotype DX testing in early-stage breast cancer."

The manuscript has been evaluated by external reviewers. Overall, this study addresses an important and clinically relevant topic and provides valuable real-world evidence regarding the implementation of Oncotype DX testing in routine multidisciplinary breast cancer care. The strengths of the work include the use of a consecutive patient cohort, a detailed description of the implementation pathway, and the assessment of both clinical and economic outcomes within a publicly funded healthcare system. The manuscript also provides important national data from Finland, where real-world evidence regarding genomic assay implementation remains limited.

The reviewers raised concerns regarding patient selection, the absence of a non-Oncotype comparator cohort, and the assumptions underlying the economic analysis. After considering the reviewers' comments and the authors' responses, I am satisfied that the primary objective of the study is to evaluate the real-world implementation and clinical impact of Oncotype DX testing rather than to further validate the assay itself. The clarifications provided in the revised manuscript have strengthened the methodological transparency and interpretation of the findings.

Before acceptance, I encourage the authors to further emphasize the limitations of the economic analysis. In particular, the projected societal cost savings appear to be driven largely by assumptions regarding productivity losses and duration of sick leave among chemotherapy-treated patients. As patient-level economic data were not available, several cost estimates were necessarily based on assumptions and average unit costs. While the sensitivity analyses are helpful and support the overall direction of the findings, the Discussion would benefit from a more explicit acknowledgement that the magnitude of the estimated savings may vary depending on real-world employment patterns, return-to-work timelines, and healthcare utilization. Accordingly, conclusions regarding economic benefit should be presented as projected estimates under the assumptions of the model rather than as directly observed cost savings.

Subject to these minor revisions, I believe the manuscript makes a useful contribution to the literature and will be of interest to clinicians, healthcare administrators, and policymakers involved in personalized breast cancer management.

I therefore invite the authors to submit a revised version addressing the remaining minor points.

Sincerely,

Academic Editor

[Note: HTML markup is below. Please do not edit.]

[NOTE: If reviewer comments were submitted as an attachment file, they will be attached to this email and accessible via the submission site. Please log into your account, locate the manuscript record, and check for the action link "View Attachments". If this link does not appear, there are no attachment files.]

To ensure your figures meet our technical requirements, please review our figure guidelines: https://journals.plos.org/plosone/s/figures

You may also use PLOS’s free figure tool, NAAS, to help you prepare publication quality figures: https://journals.plos.org/plosone/s/figures#loc-tools-for-figure-preparation.

NAAS will assess whether your figures meet our technical requirements by comparing each figure against our figure specifications.

# Author comment: Author response to Decision Letter 2

**DOI:** 10.1371/journal.pone.0353941.r004

27 Jun 2026

We are grateful for the constructive comments provided during the review process, which have helped us to further improve the clarity of the manuscript. In this second revision, we have addressed the remaining points raised in the Discussion, with particular attention to clarifying the interpretation of the economic analysis and explicitly acknowledging the assumptions and uncertainties underlying the cost impact estimates.

Attachment Submitted filename: Response_to_Reviewers_auresp_2.docx

# Editor report: Decision Letter 2

**DOI:** 10.1371/journal.pone.0353941.r005

**Authors:** UDAYAN BHATTACHARYA, UDAYAN BHATTACHARYA, UDAYAN BHATTACHARYA

1 Jul 2026

Real-world implementation and cost impact analysis of Oncotype DX testing in early-stage breast cancer

PONE-D-25-55193R2

Dear Dr.  Mäenpää,

We’re pleased to inform you that your manuscript has been judged scientifically suitable for publication and will be formally accepted for publication once it meets all outstanding technical requirements.

Within one week, you’ll receive an e-mail detailing the required amendments. When these have been addressed, you’ll receive a formal acceptance letter and your manuscript will be scheduled for publication.

An invoice will be generated when your article is formally accepted. Please note, if your institution has a publishing partnership with PLOS and your article meets the relevant criteria, all or part of your publication costs will be covered. Please make sure your user information is up-to-date by logging into Editorial Manager at Editorial Manager® and clicking the ‘Update My Information' link at the top of the page. For questions related to billing, please contact billing support.

If your institution or institutions have a press office, please notify them about your upcoming paper to help maximize its impact. If they’ll be preparing press materials, please inform our press team as soon as possible -- no later than 48 hours after receiving the formal acceptance. Your manuscript will remain under strict press embargo until 2 pm Eastern Time on the date of publication. For more information, please contact onepress@plos.org.

Kind regards,

UDAYAN BHATTACHARYA

Academic Editor

PLOS One

Additional Editor Comments (optional):

Dear Dr. Mäenpää,

I am pleased to inform you that your manuscript, "Real-world implementation and cost impact analysis of Oncotype DX testing in early-stage breast cancer" (Manuscript Number: PONE-D-25-55193R2), has been formally accepted for publication in PLOS ONE.

The revisions made to the manuscript have satisfactorily addressed the remaining points raised during the review process. We appreciate your diligent work and responsiveness throughout the evaluation of your paper.

The PLOS ONE production team will be in touch with you shortly regarding the final formatting requirements, proofs, and publication schedule.

Thank you for choosing PLOS ONE to publish your research. Congratulations on the acceptance of your manuscript.

Sincerely,

Dr. Udayan Bhattacharya

Academic Editor

PLOS ONE

Reviewers' comments:

# Editor report: Acceptance letter

**DOI:** 10.1371/journal.pone.0353941.r006

**Authors:** UDAYAN BHATTACHARYA, UDAYAN BHATTACHARYA, UDAYAN BHATTACHARYA

PONE-D-25-55193R2

PLOS One

Dear Dr. Mäenpää,

I'm pleased to inform you that your manuscript has been deemed suitable for publication in PLOS One. Congratulations! Your manuscript is now being handed over to our production team.

At this stage, our production department will prepare your paper for publication. This includes ensuring the following:

* All references, tables, and figures are properly cited

* All relevant supporting information is included in the manuscript submission,

* There are no issues that prevent the paper from being properly typeset

You will receive further instructions from the production team, including instructions on how to review your proof when it is ready. Please keep in mind that we are working through a large volume of accepted articles, so please give us a few days to review your paper and let you know the next and final steps.

Lastly, if your institution or institutions have a press office, please let them know about your upcoming paper now to help maximize its impact. If they'll be preparing press materials, please inform our press team within the next 48 hours. Your manuscript will remain under strict press embargo until 2 pm Eastern Time on the date of publication. For more information, please contact onepress@plos.org.

You will receive an invoice from PLOS for your publication fee after your manuscript has reached the completed accept phase. If you receive an email requesting payment before acceptance or for any other service, this may be a phishing scheme. Learn how to identify phishing emails and protect your accounts at https://explore.plos.org/phishing.

If we can help with anything else, please email us at customercare@plos.org.

Thank you for submitting your work to PLOS ONE and supporting open access.

Kind regards,

PLOS ONE Editorial Office Staff

on behalf of

Dr. UDAYAN BHATTACHARYA

Academic Editor

PLOS One
